# A genre-based approach in the secondary school English writing class: Voices from student-teachers in the teaching practicum

**DOI:** 10.3389/fpsyg.2022.992360

**Published:** 2022-09-06

**Authors:** Chang Liu, Meihua Chen

**Affiliations:** School of Foreign Languages, Southeast University, Nanjing, China

**Keywords:** genre-based approach, EFL writing, writing instruction, teacher knowledge, student-teacher, teacher education

## Abstract

While the genre-based approach (GBA) has assumed increasing prominence in discussions of writing pedagogy for diverse classrooms, little is known about how secondary school student-teachers understand and adopt genre pedagogies in the English as a foreign language (EFL) writing class. Based on the data from semi-structured interviews and teaching materials, this study examined Chinese EFL student-teachers’ knowledge and use of genre-based writing instruction (GBWI) during the teaching practicum and explored the challenges they encountered in enacting it. The findings demonstrated that teacher informants showed some familiarity with genre pedagogies, especially in terms of scaffolding the linguistic features and semantic patterns in the focused genres. However, they were generally confused over the connection between language, content, and context, and their GBWI practice scarcely involved the explicit teaching of the linguistic and semantic choices for a specific audience and context, which gave rise to some perceived tensions in the teaching reality. Further probing has revealed the complex interplay between Chinese EFL student-teachers’ professional knowledge, perceived difficulties, and genre instructional practice in the secondary school writing class. The study concludes with practical implications for the student-teachers’ professional development of effective GBA.

## Introduction

Over the past 20 years, the significance of genre-based approach (GBA) has gained traction in scholarship related to the second and foreign language writing pedagogies (Troyan, 2016; Cheng and Chiu, 2018; Accurso and Gebhard, 2021; Kindenberg, 2021; Troyan et al., 2022). Though the discussion of genre traditions varies in terms of three schools, Systemic Functional Linguistics, English for Specific Purposes, and North American New Rhetoric (Hyon, 1996), scholars generally agree on defining it as a “socially recognized way of using language in response to the reoccurring communicative situations” (Worden, 2019. p. 1). GBA, accordingly, places a premium on the teaching of meaning-making patterns in relation to the context of language use (Hyland, 2007). As Tardy (2017) neatly puts it, the explicit focus on the relationships between purpose (context), audience (situation), and form (linguistic choice) of the focused genre stands at the core of GBA, which can be achieved through the “inductive, discovery-based” (Hyland, 2007, p. 150) approaches to learning.

Against the backdrop of the prevalence of GBA, the Teaching and Learning Cycle (TLC) has been adopted as the typical genre instructional model in school-based literacy education (e.g., Yasuda, 2011, 2015; de Oliveira and Lan, 2014; Humphrey and Macnaught, 2016; Abdel-Malek, 2019, 2020). Recent studies have proven the effectiveness of TLC in improving students’ linguistic accuracy, lexical diversity, content development, and rhetorical organization in the process of learning writing (Chen and Su, 2012; Caplan and Farling, 2017; Huang and Zhang, 2020; Zhang and Zhang, 2021). Other studies, however, have seen in a negative light, arguing that the imposition of generic rules in TLC may restrict students’ creative language use, especially for the learners of higher language proficiency (Hermansson et al., 2019; Moore, 2019).

Regardless of the different stances on the advisability of GBA, compared with other EFL writing instructional approaches like the product-oriented approach, which accentuates students’ accurate use of vocabulary, syntax, and cohesive devices in writing (Jiang et al., 2021), and the process-oriented approach, which engages students with a recursive process of prewriting, drafting, revising, editing, and publishing, GBA can help students not only notice the linguistics features and rhetorical structures of a certain text, but also understand why they are writing (purpose), who they are writing for (audience), and how to write (organization) in order to realize particular communicative purposes of the text (Chen and Su, 2012). To achieve the desirable learning outcomes, researchers have generally agreed on the centrality of teacher knowledge, that is teachers’ “thinking about teaching and learning that guides their classroom decisions and actions” (Sun and Zhang, 2022. p. 2), on the success of genre pedagogies (Worden, 2018a,^2019^; Kindenberg, 2021). On this note, the studies concerning teachers’ knowledge base, conceptions, and cognitions of genre instruction have mushroomed, evincing these psychological factors as imperceptible but crucial indicators of engaging genre approaches and can also be supported through professional education or courses (Gebhard et al., 2013; Brisk et al., 2021; Matruglio, 2021; Rosa and Hodgson-Drysdale, 2021; Nazari and Oghyanous, 2022).

Given its recency, little research has investigated teachers’ GBWI experience by heeding the voice of pre-service teachers, who may encounter a variety of challenges related to teaching, classroom management, and interaction with students in the process of trying on the genre approaches (Yuan and Lee, 2016), which has a direct bearing on their future instructional decisions. Even less explored is the awareness and deployment of GBA by EFL secondary school student-teachers. This line of research is particularly necessary since to learn the student-teachers’ experience, concerns and struggle from an internal perspective, we can have a nuanced and contextualized view of how genre teaching is adopted in local schools, tracing the roots of the perceived obstacles in order to fix them and help GBWI achieve some prominence in the EFL secondary school education. To respond to this lacuna in the literature, this small-scale qualitative study explores eight Chinese EFL secondary school student-teachers’ knowledge and use of GBWI in the teaching practicum. By doing so, it is hoped that a firm contextual grounding can be established to support teachers’ enactment of GBA as part of their writing pedagogies across the Chinese EFL secondary school contexts and beyond. Specifically, this study answers the following three research questions:

RQ1: What is secondary school student-teachers’ understanding of GBWI?

RQ2: What strategies did student-teachers employ in adopting GBWI?

RQ3: What challenges did student-teachers encounter in adopting GBWI?

## Literature review

### Genre-based approach: Rationale and challenges

In this study, teachers’ knowledge of genre as well as their application of it to teaching is underpinned by SFL genre theory, given that it foregrounds the explicit instruction of micro-level language choices to construct meaning (Martin and Rose, 2008; Yasuda, 2015) and is empirically appropriate for novice writers in secondary schools (de Oliveira and Lan, 2014; Ramos, 2014; Huang and Zhang, 2020). According to Martin and Rose (2008), the defining feature of SFL genre theory lies in the dialectical relations between language and context. To be more specific, the form of language is mediated by the context of language use, including both the context of culture which decides the communicative purpose of written texts (genre) and the context of situation where the textual meaning-making occurs (register). There are three register variables, what was happening (field), how participants interact (tenor) and through which means (mode). The three components both dictate and are realized through the use of language, which is correspondingly chosen to construct three meanings of texts: the ideational (i.e., how the content is expressed), the interpersonal (i.e., how the relations between the reader and the writer are addressed and attitudes are expressed), and the textual (i.e., how the text is organized and cohesion is established) (Martin and Rose, 2008; Abdel-Malek, 2020).

To make the form-meaning-context connections accessible to learners (Hyland, 2007), SFL genre pedagogies have been advanced considerably based on the TLC (Rothery, 1996), which argued that language learning occurs, in principle, through scaffolding (i.e., teacher-supported learning) and collaboration (i.e., peer interaction) in the context of the shared experience of working with texts (Martin and Rose, 2008). In this sense, the teaching-learning process is enacted as a cycle of three consecutive stages, to build the context and field of the text (modeling), guide students to analyze the sample texts to deconstruct the discoursal stages and the organization and language choices within each stage (joint deconstruction), and let students construct meaning to perform the thematic patterns and grammatical features of a specific genre (independent construction) (Rothery, 1996). In doing that, teachers are expected to give explicit guidance at the initial stage, but the support should be strategically diminished as learners progress until they become independent producers of a range of genres (Derewianka, 2003). As such, the TLC provides a pedagogical tool for teachers to support students in learning to write effective school texts (de Oliveira and Lan, 2014; Shum et al., 2018).

Beyond the theoretical consideration, GBA has also been widely practiced to teach genres like recounts (Abdel-Malek, 2019), summary (Chen and Su, 2012; Yasuda, 2015), science writings (de Oliveira and Lan, 2014), persuasive and argumentative essays (Ramos, 2014; Moore, 2019; Huang and Zhang, 2020; Zhang and Zhang, 2021) in a range of educational contexts. Whilst there is no lack of empirical findings supporting that GBA can help students notice the discoursal formula and linguistic features of different genres (Yasuda, 2011, 2015; Abdel-Malek, 2019, 2020), use functional metalanguage as cognitive tools to mediate meaning-making (Negretti and Kuteeva, 2011; Moore and Schleppegrell, 2014; Iddings, 2021), and finally integrate various facets of genre knowledge to enhance the overall writing performance (de Oliveira and Lan, 2014; Humphrey and Macnaught, 2016), the adoption of GBA is considered risky and demanding for teachers. Challenges can stem from the integrated focus of genre-specific conventions and contextual awareness (Johns, 2011; Yayli, 2011), the conflicting nature of genres as both stable and flexible entities (Worden, 2018b; Kindenberg, 2021), the place of language in genre instruction (Cheng, 2019; Li et al., 2020), and the scaffolding of metalinguistic concepts and metacognition to facilitate the actual use of genre (Negretti and Kuteeva, 2011; Wette, 2017; Iddings, 2021). These obstacles are even complicated by internal factors related to teachers themselves, such as their dual roles in preparing and judging students in summative tests and simultaneously facilitating their writing competence in the long term (Lee, 2012), and importantly, their lack of expertise to use genre pedagogies flexibly in line with the local needs, especially for the inexperienced teachers (Tardy, 2017; Tardy et al., 2018; Li et al., 2020). To better engage in GBWI, it is necessary to understand teachers’ conceptions and practices in the first place.

### Teachers’ knowledge and practice of genre-based writing instruction

Given that the explicit teaching of the genre in GBA is highly dependent on teacher knowledge (Worden, 2018a,b; Kindenberg, 2021), writing teachers, the genre experts in the classroom, are expected to be “conscious of the genre decisions they make and what those decisions will teach students” (Devitt, 2009, p. 339). In this sense, teachers with adequate genre expertise are in a better position to give pedagogical intervention, who can not only offer content, linguistic and structural scaffolding through genre analysis and modeling but also connect these generic features to the writing purposes and contexts to raise students’ meta-awareness (Hyland, 2003, 2007; Johns, 2011; Choi and Wong, 2018; Worden, 2018b; Kindenberg, 2021). Nevertheless, previous research also identified the practical challenges encountered by unprepared teachers to balance the discursive regularity and rhetorical flexibility in the genre approach (Fisher, 2006; Gebhard et al., 2013; Moore, 2019). Being aware of the stable conventions in the focused genres, some teachers may pinpoint generic forms as transferable rules to enable “students to learn with confidence” (Johns, 2011, p. 58) and better prepare for the tests (Fisher, 2006). However, since it disconnects the discernable structural and lexical features of texts from social contexts, students are also restricted in developing rhetorical and genre awareness to conceive the influence of audience and context on language use and to express and create ideas in text generation (Hyland, 2003; Choi and Wong, 2018; Moore, 2019). Therefore, the conflicting nature of genre as a simultaneous stable and flexible entity (Worden, 2018b; Kindenberg, 2021) is closely related to the dual teaching focus on genre-specific knowledge and genre awareness, which perplexes teachers a lot or even is overlooked by teacher cognition (Johns, 2011; Yayli, 2011).

In FL writing contexts, the few studies on teachers’ use of GBA have revealed how genre instructional practices have been adapted to the situated needs (Li et al., 2020; Rosa and Hodgson-Drysdale, 2021). According to Brisk et al. (2021), as the teacher learned SFL theory and pedagogy, his GBA departed from a faithful recreation of the TLC to cater to himself and students’ needs. Similarly, Matruglio (2021) suggested that teacher participants who attended the same training seminar showed different uptake. One teacher enthusiastically adopted joint writing from TLC without using the metalanguage, whereas the other did not integrate joint writing but engaged in the explicit teaching of metalanguage and clause-level grammar. Though reshaping the praxis differently based on different instructional contexts, both teachers reaped positive outcomes in terms of student confidence and ability to write for examination. Gebhard et al. (2013) also noted that teachers’ use of prescriptive templates was a response to assessment and system, which urged teachers to downplay variation and make genres more accessible, the finding supported by Fisher (2006). Therefore, it can be noted that teachers’ GBA practices are mediated by an array of contextual factors, like the availability of training and development resources (or lack thereof), student interactions, and dominant assessment culture, among others (e.g., Lee, 2012; Gebhard et al., 2013; Worden, 2018a; Troyan et al., 2022).

The aforementioned literature suggests the complexity of teachers’ GBA practices, mediated by both internal teacher cognition and the external teaching environments. Notwithstanding a plethora of studies on teachers’ development of genre-related knowledge and praxis, always afforded by professional coursework and teacher education projects in English-specking contexts (Gebhard et al., 2013; Worden, 2018a,b, 2019; Accurso and Gebhard, 2021; Sembiante et al., 2021), little is known about how student-teachers conceptualize and enact genre pedagogies in the secondary school EFL writing class. A focus on student-teachers’ knowledge and practice of genre instruction in the teaching practicum is important because as novice teachers, they are in a crucial stage of forming and reforming the teaching beliefs and practice systems, and might be struggling with all sorts of practical problems because of their immature professional identity (Yuan and Lee, 2016; Yu et al., 2020). Therefore, the current study seeks to expound on how GBWI has been understood and enacted by student-teachers in their teaching practicum by taking up Tardy’s (2017), p. 174) calling to illuminate the “challenges that teachers face” in genre instructions. Such a study can provide an empirical ground to clarify the tensions teachers are encountered in enacting GBWI in secondary school education, thereby guiding the design and improvement of teacher education programs to help prospective teachers solve, or at least minimize the obstacles and better embrace GBA as a significant aspect in EFL contexts like China.

## Materials and methods

### Participants and contexts

According to Patton (2002), purposeful sampling “is to select information-rich cases whose study will illuminate the questions under study” (p. 46). In the current study, eight student-teachers from two public normal universities familiar to the authors were recruited purposefully, so we can build rapport with them to get more reflective views. Located in Jiangsu and Hebei respectively, two universities are differentiated in terms of school rankings, requirements for entrance, and faculty qualifications to represent regional and demographic heterogeneity, given that the educational resources in China are distributed unequally across the country (Jiang et al., 2021). Five participants were from undergraduate language teacher education programs, who had participated in a teaching practicum in the fifth/sixth semester, and taught English for over 10 weeks in the field schools. Before the practicum, they had been exposed to a series of language proficiency and linguistic courses, like English Writing, Linguistic, and Lexicology, as well as language teacher education courses, like Approaches to Language Teaching, Curriculum and Syllabus Design, and Educational Psychology. The other three participants were learning for their Master’s degrees at the university in Jiangsu. Though in different majors, they were all assigned to teach at the local secondary schools for over 10 weeks. According to the program arrangement, the local schools were required to assign a mentor, an experienced teacher to each student-teacher to support and evaluate their performance. University supervisors also paid regular visits to student-teachers in different field schools and provide professional guidance. The interviewees’ profiles, including their ages, educational backgrounds, and majors are presented in Table 1. All participants are assigned pseudonyms to protect privacy.

**TABLE 1:** Participant profiles.

Teacher	Age	Degree	Major	Target students	Location
Amy	24	MA	Applied linguistics	Grade 9th	Jiangsu
Lisa	25	MA	Translation	Grade 10th	Jiangsu
Molly	24	MA	Applied linguistics	Grade 9th	Jiangsu
Candy	22	BA	English education	Grade 11th	Jiangsu
Kitty	23	BA	English education	Grade 11th	Jiangsu
Annie	25	BA	English literature	Grade 10th	Hebei
Linda	23	BA	English education	Grade 8th	Hebei
Hanna	22	BA	English education	Grade 10th	Hebei

### Data collection

Data for the study was mainly collected *via* in-depth, semi-structured interviews. Three broad topics guided the interviews: (1) the respondents’ understanding and knowledge related to genre and GBA, including but not limited to its definition, principles, teaching focus, instructional procedures, and rationales; (2) the use of GBA in writing instruction; (3) the practical challenges and obstacles encountered in implementing GBWI in different teaching contexts (see Supplementary material for the specific interview questions). The first topic focused on teachers’ conceptions of genre and its application to teaching writing, intending to elicit student-teachers’ overall knowledge of GBA. The second was concerned with the concrete strategies teachers employed to enact GBWI and the third one dealt with their perceived challenges while implementing it in their own contexts. During the interview, special attention was given to the details and reasons for the teaching decisions, so that we could know not only how but also why GBWI was conceived and accordingly enacted in certain ways.

After seeking informed consent, one or two rounds of online interviews were conducted with each informant. Each interview lasted for around 40 min and was conducted in Mandarin. The first interview aimed at obtaining participant teachers’ personal information and general responses to the intended questions, and the follow-up interviews elicited their clarification on some informative responses. For instance, at the end of or after the first interview, respondents were asked for sample teaching materials and student texts, which served as artifacts to prompt their recall and detailed description of GBWI practices in the second round of interviews. We also invited some teachers to expound on some informative responses they raised in the first interviews. All interviews were recorded and then manually transcribed for analysis.

### Data analysis

**FIGURE 1:**
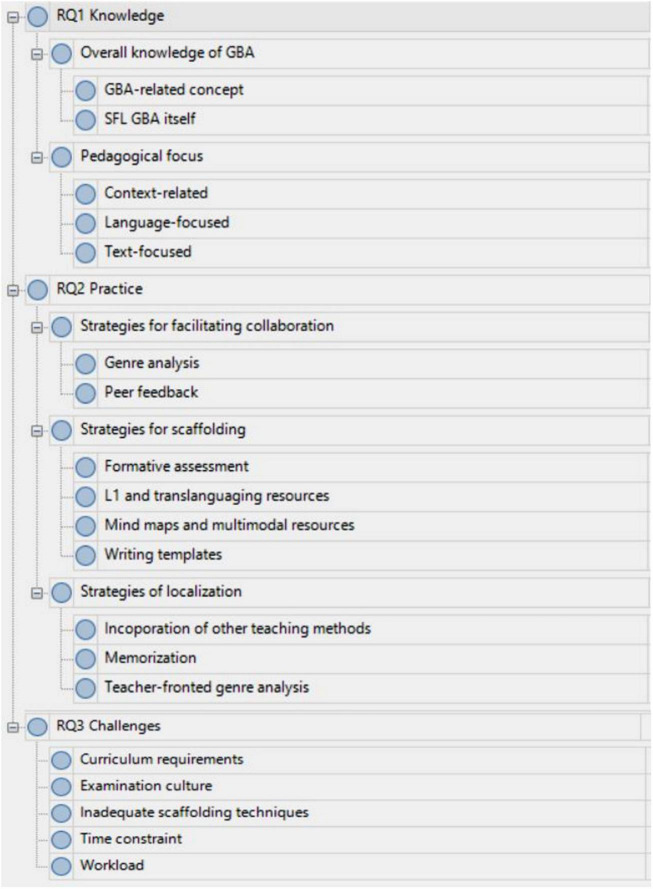
A screenshot of the coding system in Nvivo. Reproduced with permission.

Following Braun and Clarke (2006)’s guidelines, interview data were imported into NVivo 11 Pro software (QSR International, 2017) for thematic analysis. Sample teaching materials were utilized as Supplementary Data. The analysis involved five stages. First, the first author familiarized data by reading through the interview transcripts to obtain a general sense. Second, open coding was performed to scrutinize teacher responses, identify excerpts related to the three research questions and assign codes to them by using native codes. For example, “translate the difficult concepts into Chinese,” and “show students the mind map” were coded as “translating to L1” and “using mind maps” since they were concerned with the second research question, the teaching strategies to implement GBA. Then codes from different data sources, interviews, and the collected artifacts, were compared within and across different cases to converge into themes through the recursive process of shuttling between the collected data and existing literature. For example, drawing upon the existing studies about teachers’ use of GBA (e.g., Gebhard et al., 2013; Tardy, 2017; Cheng and Chiu, 2018; Worden, 2018a,b, 2019; Li et al., 2020; Kindenberg, 2021), the codes “translating to L1,” “using mind maps” were categorized into the theme of strategies for scaffolding, together with the other two themes: the strategies to facilitate collaboration and strategies of localization. In the fourth stage, the themes were inductively grouped into three major categories, corresponding to the research focus of teachers’ understanding of GBA (i.e., knowledge), implementation of GBA in teaching writing (practices), and the perceived constraints on their GBWI practices (challenges). Lastly, to ensure the validity and trustworthiness of the findings, the second author reviewed the themes and codes, and a final theme list was generated until the disagreements were resolved to reach a consensus after rounds of discussion (see Figure 1 for the coding system from NVivo). The first author also used the reflective journals to review the themes, achieving the intra-coder agreement and internal consistency of the coding process. The preliminary findings in interpreting and presenting the main findings were revised accordingly. Member checking was performed with two participants.

## Results

The findings are divided into three sections in accordance with three research questions. In each subsection, findings are discussed in detail with the translated interview excerpts and relevant teaching materials.

### Student-teachers’ knowledge of genre-based writing instruction

By and large, our respondents perceived themselves as inadequately informed of the principles and implementational procedures of GBA, and had a unanimous agreement on the necessity of professional improvement in this regard. Only three participants (Molly, Candy, and Kitty) were acquainted with the term GBWI *per se*, either from their supervisors by attending seminars or from reading professional literature. However, they were still unsure that they took practical command of it in real teaching, as shown in the quote below:

I have learned about what GBWI is, but that does not mean I have a full grasp of how to implement it [.]. In my teaching practice, I might adopt a few aspects of it, like leading students to the key elements of narrative texts. I am unsure whether there is anything else out of my command (Kitty).

The rest of the participants expressed their different degrees of familiarity with some GBA-related notions, like genres, and text types, and Lisa also drew on the concept of genre-based reading instructions, which she learned from the internship as it was widely adopted at the local high school. Most participants defined the genre as a category or type of texts, with such discrete features as the “expressed content” (e.g., Cindy), “word choices” (Annie and Lisa), and “structures and formats” (Amy and Hanna) figuring prominently in their conceptualization. They also tended toward school texts like narratives, and practical and argumentative writings when providing examples of genres. When asked about the main criterion for differentiating genres, Candy, a student-teacher at a well-resourced foreign language middle school in Jiangsu, responded that,

“Narratives and argumentative texts have different writing purposes and textual characteristics, so I think they are two different genres. The words and sentences used in both types are different. There are more emotional, background, character, and psychological descriptions in narrative texts, but argumentative texts focus on elaborating and refuting opinions” (Candy).

It is worth mentioning that according to Candy’s conceptualization of genre, writing purpose referred to different content or textual meaning the authors conveyed. On that basis, “the discoursal patterns of a certain genre, along with how it was constructed” (Candy) were perceived as the pedagogical focus of GBWI, and the teaching objective was to “raise students’ genre awareness,” which, in her mind, was a synonym for genre-specific textual knowledge, that is, “when students approach the writing task, they should know what type of text they will compose at the first sight, how about its text structure, language features, and writing purposes” (Candy). This aim could be achieved by “drawing upon the exemplars of a given genre, analyzing its organizations and thematic structures, letting students imitate the exemplars, revise the texts and finally produce works independently” (Candy).

In the meantime, four student-teachers, Lisa, Molly, Annie, and Linda also had a micro-focus on language choices, accentuating the different use of vocabularies, chunks, and phrases in analyzing texts of different genres. For instance, in Linda’s practice of GBA to teaching recommendation letters, she “showed students two or three sample texts. Since there are some common words and fixed expressions at the beginning and the end of the letters and in thesis statements, I highlighted them to provide students with enough input” (Linda).

**FIGURE 2:**
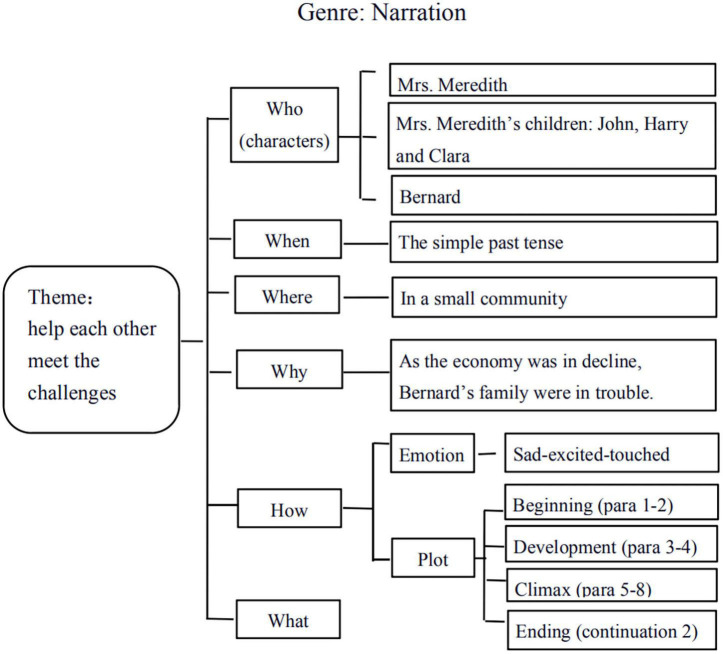
The analytic framework for narrative texts (PowerPoint slide from Candy). Reproduced with permission.

Not surprisingly, with little reference to the context of writing and language use in defining the genre, the interviewees scarcely involved the interconnections between rhetorical situations and linguistic features of texts in their self-reported GBWI practices to raise students’ contextual awareness. Annie, in this regard, referred to the social nature of genre “as a means for communication in certain contexts” when talking about the benefits of GBWI to enhance students’ writing motivation, but she did not explicate how to enact teaching to make such social function of genre accessible to students. Similarly, the presence of social contexts can be traced in Candy’s teaching of how to write a continuation of the given narrative text. Based upon the generic framework which provided the six elements of narrative texts (who, when, where, why, how, what) as the settings of storytelling (Figure 2), she indicated,

“I asked my students to consider these elements while writing, for example, the given characters should also appear in the produced texts, and their writings must conform to the time and place of the story, reflect the main characters’ emotional change, echo the theme, and be well-organized in terms of plot” (Candy).

It can be seen that in drawing students’ attention to the rhetorical settings in genre-based writing, Candy seldom explicitly mentioned the interconnections of particular contexts with the appropriate language forms, as noted by the scant refer to such specific linguistic features as “the use of simple past tense” (Figure 2) within the frame of communicative contexts.

### Student-teachers’ use of genre-based writing instruction

#### Strategies for scaffolding

In the self-reported GBWI practices, nearly all (seven out of eight) respondents foregrounded how they made generic features accessible to students. To stimulate the students’ notice and provide more comprehensible input, the student-teachers employed a variety of scaffolding techniques, ranging from using mind maps and multimodal resources to drawing on L1 or translanguaging to explain abstract concepts like grammatical features. The use of translanguaging resources was represented in Linda’s description of teaching how to write diaries at a junior high school in Hebei:

“Since the students had learned how to write diaries in their Chinese class, and they also wrote diaries in the daily life, I introduced the concept of diary in Chinese, and let the students compare it with English diary to facilitate their understanding and deepen their impression” (Linda).

As regards the stage of text production, writing frames were widely employed by six respondents to provide skeletal outlines for students to organize ideas within the macro structures related to certain genres. The use of templates sometimes took the form of controlled practice, in which students were asked to “imitate the sentence patterns of sample diary in the textbook by adding or changing some words” (Linda) since the writing tasks dealt with similar topics with the given sample. More teachers like Hanna, Kitty, and Amy, nevertheless, scaffolded the writing process in a less intrusive way, asking students to generate ideas by themselves. In these freer activities, the student-teachers also offered support by “explicitly reminding students of employing generic moves and using the collected sentence patterns from the model texts whenever there was a chance” (Kitty). Various forms of assessment were also adopted to provide scaffolding, like showing students assessment criteria before the independent writing (Lisa) and engaging them in multiple turns of peer and self-assessment (Hanna and Annie). However, the focus of assessment criteria was rather general with a lack of the explicit inclusion of genre-specific dimensions, as demonstrated in the response below,

“The assessment criteria at first addressed language errors because the text must be written in the past tense, and the grammatical structure of time adverbial clause learned in this topic should also be used correctly; second, whether the content revolved around the topic and theme; third, logic, which means the article should be well-organized by the linking words” (Hanna).

#### Strategies for facilitating collaboration

Compared to teacher-supported scaffolding, another major notion epitomized in GBA, collaboration, seemed to be less adopted and mentioned by five responded student-teachers. Lisa, Candy, and Linda reported asking students to analyze and compare several text samples in small groups to deduce the linguistic and structural features of certain genres. For example, Linda instructed on the generic features of recommendation letters by giving students three sample texts and asking them to discuss their common features. In doing that, she directed the students’ attention to some specific dimensions, like “How the first text is written in terms of format and language used at the beginning, middle, and end of the letter? Do other texts share these features?” (Linda). In the following stage of acting on the newly acquired genre knowledge and literacy skills in producing texts, collaborative writing was adopted by Candy, Annie, and Kitty occasionally, which was contingent on the time schedule and students’ English proficiency. As raised by Kitty,

“Due to the time constraint, I’ve just used collaborative writing several times. It takes nearly 40 min for the students to write an article, leaving little time for discussion. If time permitted, I would use more collaborative writing to let the students brainstorm and give mutual help, which is expected to work more effectively than individuals write separately” (Kitty).

Peer reviewing, another major form of collaboration was also employed by Lisa and Annie, who asked their students to correct grammatical errors, highlight the well-written sentences and select several exemplars to share with the whole class (Lisa).

#### Strategies of localization

Noteworthy, it has been found that student-teachers’ GBWI strategies were localized based on their understanding of the classroom realities. Teaching at a senior high school in Jiangsu where the students were overburdened by learning for the high-staking examinations, Lisa asked her students to memorize chunks or recite sample texts, so students can be relieved to “apply them to writing in examinations automatically without further intentional thinking.” The other four respondents also required students to either imitate model texts or improve their drafts based on multiple turns of peer and teacher feedback and final memorize the products by heart. Simultaneously, five teachers opted for the teacher-fronted genre analysis, asking students to deduce key genre elements (e.g., Candy and Kitty) and language use features (Lisa, Annie, and Linda) from sample texts instead of discovering them inductively. Candy, by referring to Figure 2, explained that in her real classroom teaching, the backbone of this mind map (i.e., the six elements of narrative texts including who, when, where, why, how, and what) was provided before the student discussion:

“Since I am unsure whether they were familiar with the narration, and I think it is necessary to direct their attention to these specific aspects, otherwise the students might be lost, feeling that they spent so much time on the discursive discussion yet acquired little concrete knowledge” (Candy).

**FIGURE 3:**
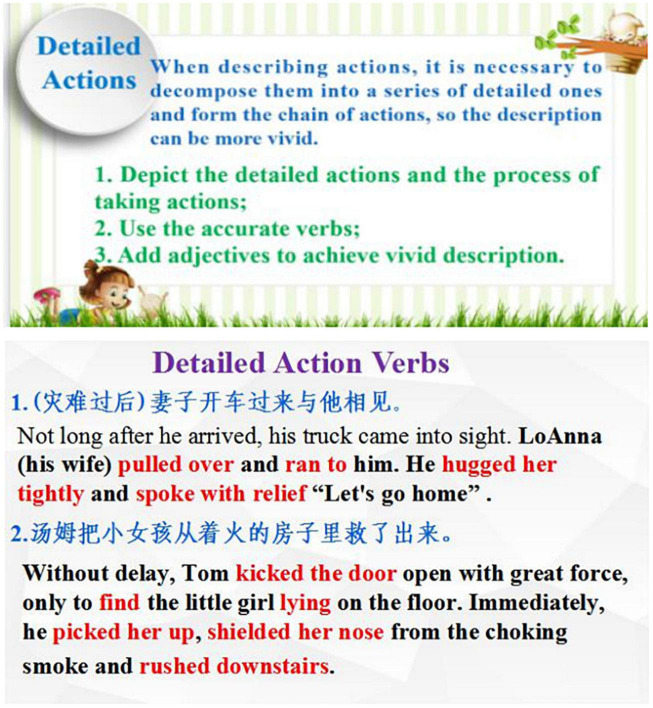
The teacher-fronted genre analysis (PowerPoint slides from Annie). Reproduced with permission.

In the case of Annie, she dominated genre analysis mainly for the sake of explaining the focused language choices and giving the controlled exercise (e.g., by filling in the blanks to notice the use of verbs, as illustrated in Figure 3). Furthermore, the reported GBWI practices looked differently between student-teachers in Jiangsu and Hebei. As mentioned above, teaching in an under-resourced middle school in Hebei, Linda used controlled exercise to help student digest or memorize the fixed sentence structures in narrative writings, given that the task of changing single words or phrases were perceived within the students’ capacity. However, the teachers from Jiangsu like Candy and Amy gave more freedom to students to express their own ideas due to the fact that the students were believed as more advanced learners able to independently write complete and accurate sentences. Strikingly, even with little professional experience, Molly already had the awareness to incorporate elements of innovative teaching methods into GBA to solve real teaching problems, like using the “motivating” stage from the Production-Oriented Approach, a localized teaching method, to stimulate the students’ learning motivation at the beginning of the class. Such attempts were not found in the respondents from Hebei, who were more preoccupied with making the language points, such as the use of action verbs (Annie, as shown by Figure 3) and adverbial clauses (Linda) accessible to students.

### Challenges encountered in implementing genre-based writing instruction

With respect to the third research question, the lack of professional expertise to scaffold students based on their current proficiency was identified as the major challenge, as typified in the quote below:

“One major challenge for me is to provide input that makes sense to students, to let them understand what I am instructing. When I was a middle school student, the teachers just gave us model texts and let us memorize them [.]. Now I am a teacher, I really want to use effective methods to teach students how to write, so I observed the writing class instructed by the experienced teachers, but since I worked alone, the workload for preparing for a class from scratch was huge for me, and it was disappointing that the students still had little response. Their eyes showed that they did not understand what I was doing” (Annie).

It can be noted from the above quotation that the practical challenge of scaffolding was intensified by the heavy workload in the absence of collaboration from the teacher community. In a similar vein, Kitty also expressed her diffidence in conducting genre analysis to explicate linguistic concepts (like register, style) and conventional features of a certain type of text, which can be partially ascribed to the time constraints for students to analyze jointly and thoroughly: “If I let the students discuss and then explained the features of narrative texts thoroughly, I would devote almost one entire class to genre analysis, leaving little time for other tasks” (Kitty). Therefore, it seemed unrealistic for her to lead students toward the generic features through gradual approximation, based on which students then transferred such knowledge to construct their own texts.

Syllabus and curriculum arrangement also added a burden for teachers to practice genre instruction within the tight teaching schedule. According to Candy, since the top-down curriculum standards required English textbooks and classroom teaching to address a certain number of genres in a short timeframe, usually a semester, teachers had to “shuttle between different genres, like to explore narratives in the first unit and shift to argumentative texts in the following, making it hard for both students and teachers to digest each completely.” As such, teachers’ struggle to deal with different genres restricted their utilization of GBWI, especially in senior high school contexts. Furthermore, a similar cognitive consideration is also voiced by Kitty, who worries about how to guide students’ adoptive transfer of genre-specific knowledge and literacy skills to the learning of other genre types:

“If GBA was employed, there could be too many genres for us to explore. As far as practical writing in Gaokao is concerned, the structures, writing purposes, and language use of letters and notices are different. Therefore, I am wondering how to cover the different text formats and language styles of so many genres in adopting GBWI” (Kitty).

Another major obstacle perceived by the student-teachers was the high-stakes testing culture in Chinese secondary education. More than half of respondents found it urgent and tricky to connect GBWI to students’ immediate need for grade-getting in summative examinations, which seemed more in favor of the utilitarian strategy of imitating, reciting, and reusing the model texts. According to Amy,

“I still feel that the focus of GBWI, that is the text structure and discoursal patterns, seems to be rather general to help students prepare for the tests, which ought to cover the topic, language points and the logic of point of view, among other delicate aspects of argumentation. I am unsure whether and how GBWI can benefit students in the acquisition of such specific knowledge” (Amy).

As shown by Amy’s concern, even though she believed that most of her students were of intermediate or even high proficiency who knew the schematic patterns of argumentation well, she was still perplexed about the incorporation of teaching language points in genre instruction. In her cognition, GBA was concerned with macro-level issues like content development and structure, but the summative tests above all examined students’ abilities to use the English language, namely whether they had a fluent, logical, and accurate control of English.

## Discussion

As shown in the findings, teachers’ voices are vital in understanding the actual implementation of GBA and finding the solutions to promote it. In response to the first question, we found the focal student-teachers in general were lack of familiarity with and a practical grasp of the GBA *per se*. This may be associated with their English learning experiences. As voiced by Annie, they learned how to write English texts primarily through a product-oriented approach, which accentuates the imitation of input and the accuracy of language forms. With the scarce experience in teaching writing and exploring the latest instructional methods like GBA, teacher-students in EFL secondary schools in China tend to be impacted by their learning experiences (Cheng et al., 2021). They also valued the local issues like word choices, structure, and format highly in conceiving genre pedagogies and providing examples of GBWI practices, whereas giving less weight to global issues which systematically connected the discursive features of a certain text to its audience and rhetorical purposes. Echoing the prior literature about genre teaching in both EFL and United States college composition contexts (Johns, 2011; Tardy et al., 2018), we found that “sentence-level, form-focused conventions” (Gebhard et al., 2013, p. 107) also figured prominently in EFL secondary school teachers’ conception of GBA.

As regards the second research question, our study revealed that the focal participants were prone to a structured teaching approach, valuing the model texts with discernable formats, structures, and linguistic features as the pedagogical focus to help students produce writings. Though few teachers (e.g., Candy and Annie) were cognizant of the role of social contexts in conceptualizing genre, their instructional practices still seldom involved the interactions between the aspects of context and the lexico-grammatical and semantic features of texts. This may be caused by the contextual constraints, as conflicts arose between the goal of enhancing students’ language use competence in social contexts epitomized by GBA and the expectation of getting high scores in English examinations, which indicates a lack of intrinsic motivation to improve student English proficiency in the long run. In the face of such tension, student-teachers with little experience in striking the balance between the two might find it tricky to take advantage of the essence of GBA, missing the chance of drawing on the form-meaning-context connections to let students see the meaningful use of language in social contexts. The present study supports the decisive role of teacher cognition on instructional decision-making (Borg, 2003, p. 81), and also resonates with Sun and Zhang (2022) in revealing that novice teachers might have difficulties in carrying out their teaching cognitions and knowledge consistently in instructional practices.

It has also been found that student-teachers’ GBWI practices showed varying degrees of conformity with the principles of GBA, scaffolding, and collaboration, and indicated some genre innovation to cater to the local contexts. Strategies of scaffolding were commonly adopted. Some strategies proven practical in genre analysis, like the use of mind maps (Wette, 2017), translanguaging resources (Archila et al., 2021; Troyan et al., 2021), and phrase banks to collect “multi-word sequences” (Gebhard et al., 2013; Li et al., 2020) were also adopted, representing pre-service teachers’ valuable pedagogical attempts in the implementation of GBA. One possible explanation for this is that the student-teachers in the present studies were from normal college (i.e., teacher education), and they can transfer the scaffolding techniques learned from professional courses (e.g., language teaching methods) to the use of genre pedagogies and also consciously draw from their useful EFL learning experience as students. Comparatively, collaborative learning was adopted less often due to the time constraints, which might restrict the chance for students to develop genre knowledge and literacy skills with shared consciousness as well as transfer them to subsequent independent writing (Hyland, 2007; Caplan and Farling, 2017). Noteworthy, even in the process of learning the ropes of GBA (Cheng, 2019), student-teachers were generally capable of recontextualizing the GBWI practices based on the local teaching and learning ecology, like the intentional use of controlled and less structured exercises for students with different levels of English proficiency in Hebei and Jiangsu, respectively. The strategies of localization were also evidenced by respondents like Annie and Kitty’s emphasis on “pre-while-post” writing stages and imitation of genre exemplars in the self-reported GBWI practices, given that elements of the process and product approach to teaching writing were integrated into the dominated GBA, a finding corroborates Jiang et al. (2021). In the light of Chinese EFL teachers’ “eclectic multicomponent approach to teaching writing” (Rahimi and Zhang, 2022, p. 1) to satisfy the local learning needs, future research examining the impacts of GBA on students’ writing development in similar EFL contexts should arguably be mindful of its interconnection with other approaches and adopt a holistic perspective.

Additionally, analysis of teachers’ GBWI practices in light of the visible TLC model has yielded somewhat different results. In the first place, though respondents’ specific teaching stages varied, they generally approached genre instruction through the scaffolded generic analysis of the sample texts. With the observed generic features, students were then prompted to apply them as a set of transferable rules to be obeyed in their writing. This can be accounted by the utilitarian and immediate goal of preparing students for the language proficiency-oriented standard tests. For instance, two teachers explicitly stated the piece of writing required by the tests was relatively fixed, so transferring the generic features into students’ own writing would benefit students in this regard. Another possible interpretation is the teacher-driven culture of learning in Chinese secondary school education that thinks highly of the dominance of teachers in knowledge dissemination and transformation, which prompts the respondents to lead the genre analysis and provide sufficient guidance. Further attention should also be paid to genre-based assessments, an integral part of TLC supportive of students’ increasing control of writing (Hyland, 2007; Uzun and Zehir Topkaya, 2020). The focal teacher-students have adopted formative and collaborative assessments to help students consolidate the acquired knowledge and literacy skills. Nonetheless, they seldom included genre-specific dimensions in the rubric and referred to assessment at the teaching stages, so the design of assessment seemed to be inadequately interwoven with genre instruction to foster each other. Our finding is at odds with Lee (2012)’s, which suggested the well-trained secondary English teachers in Hong Kong were capable of integrating genre-based assessment into teaching to show students the connection between tests and learning and motivate them to learn and prepare for tests. That said, to release the potentiality of genre-based assessment in EFL writing instructions, more teacher training is warranted to cover the meaningful combination of genre-based instruction and assessment.

Pertaining to the last question, the responded secondary school EFL student-teachers in China showed significant consensus in identifying the tensions discouraging them from genre instruction. The inadequate scaffolding skill was the chief challenge, compounded by external factors like time constraints, students’ varying English proficiency in the big class, and a heavy workload to prepare alone. Additionally, a small number of teachers also voiced their concern that GBA appeared to be at the variance with the curriculum arrangement, since it was unrealistic for GBA, which concentrated on one particular genre, to cover diverse genres and genre variants as required by the syllabus. Tension also arose from the dominant examination-driven culture. Accustomed to a teacher-centered instructional style and driven by the grade-getting goal, both teachers and students hesitated about the connection between language acquisition for examination and genre learning for literacy skills and were struggling to teach language points in genre instruction. These perceived challenges have echoed the previous findings on the use of genre pedagogies in mainland China (Li et al., 2020), Hong Kong (Lee, 2012; Choi and Wong, 2018), ESL in K-12 contexts in the United States (Gebhard et al., 2013) and United Kingdom (Fisher, 2006), arguing that the adoption of GBA is context-dependent, and the external obstacles beyond the control of the individual teacher exert a negative impact on their genre innovation.

The current study extends the literature on genre instruction in uncovering the complex relationships between Chinese EFL student-teachers’ knowledge, perceived difficulties, and practicing genre pedagogies in the secondary school writing class. In the first place, teachers’ professional knowledge negatively mediates the relationship between perceived difficulty and GBWI teaching. That said, student-teachers’ inadequate genre expertise might exaggerate the difficulties perceived from the physical environment in enacting teaching, such as improper textbooks, curriculum and assessment requirements, and insufficient time and training to organize genre teaching. In our cases, with a decontextualized view of genre, the unprepared teachers were unconsciously reifying genre into the “concrete facts about texts” (Johns, 2002, p. 237–238) and text production into how-to-do lists in their teaching practices, whereas the cultivation of metacognitive contextual awareness was largely overlooked. As such, they were struggling to help students address variability within the focused genre, learn the concept of genre in a motivating and creative way, and transfer genre-specific knowledge to the learning of another to attain the curriculum goals. This lends support to Hyland’s caution (2003) against an over-prescriptive use of GBA for novice teachers. It also explicates how insufficient professional knowledge creates tension in inexperienced teachers’ teaching reality, making the external factors negatively impact their genre innovation.

Second, the perceived contextual difficulties also exert a direct impact on the interaction between teacher knowledge and practice regarding GBA. As Candy indicated, even though she was cognizant of the affordance of collaborative text production, its implementation in classroom teaching is highly restricted by the time constraints. In the face of tensions from teaching reality, Annie also utilized various self-regulated strategies to improve her GBA expertise (e.g., observing classes instructed by the experienced teachers and reflecting on their own writing experience as learners), but her pathway to professional development was still impeded by the stressful teaching environments, including heavy workload, large classes, and inadequate team support, among others. That said, the perceived external constraints imposed substantial obstacles to teachers’ agency in prompting the positive interplay between GBA knowledge and practice (i.e., improving and materializing GBA knowledge through the concrete teaching practice), which might account for the possible mismatch between Annie’s and Candy’s teaching cognition and practice. This has converged with Yang et al. (2021)’s findings about the pre-service teachers’ online feedback experience, which demonstrated that the facilitative environment is necessary for student-teachers’ self-regulated learning and professional development. Given that student-teachers are still in the crucial stage of shaping and reshaping their belief system by responding to the classroom realities and “external constraints and stimulus” (Yu et al., 2020, p. 11), we call for more empathy for prospective teachers’ psychological dimension, like their confusion and frustration in implementing GBA. With challenges and their situated needs better understood, improvements in external surroundings should be made to promote student-teachers’ tenacious teaching beliefs and innovative teaching methods.

## Conclusion and implications

The current study unveiled Chinese student-teachers’ general knowledge of GBA, their control over its implementations, and the encountered challenges in the secondary EFL writing class. Findings have shown that participants predominantly reported not having sufficient and practical acquaintance with GBA, and their genre instructions, while not without merits, seldom offered students an explicit account of how the texts in target genres were written in relation to the evolving contexts, which might explain why they were sometimes confronted with tensions in genre teaching. Discussions revolve around cognitive and practical considerations of teachers’ GBWI lead to several implications that merit attention from teacher educators, administrators, and student-teachers seeking GBA professional development.

First, teacher educators are expected to collaborate with the associate teachers to provide scaffolding and reshape student-teacher’s cognition of GBA during the teaching practicum. In line with our respondents’ potential knowledge gap, some key issues should be addressed to iron out their cognitive conundrums on enacting GBWI: (a) introduce the nature of genre as a social action, explicating how language choices are systematically and dynamically linked to the writing context; (b) incorporate genre analysis in teacher training courses to model how to co-construct particular stages or functions of the texts, compare different genres to foster genre awareness (Derewianka, 2003; Negretti and Kuteeva, 2011; Yayli, 2011), and give flexible choices based on the diverse rhetorical situations of a given genre while exploring the stable generic conventions (Johns, 2011; Worden, 2018b; Kindenberg, 2021); (c) have a clear focus on language learning in genre instruction, which can be achieved through leading students toward the gradual control of phrases, patterns, or multiword sequences that signal schematic structures (Choi and Wong, 2018; Huang and Zhang, 2020; Li et al., 2020), and the assessment criteria involving genre dimensions should also be presented at the instructional stages to align genre learning goals with the standard tests.

Second, student-teachers are suggested to critically reflect on their instructional experience, including but not limited to GBA. To cope with the contextual constraints and improve genre teaching expertise, they need to prioritize self-regulation and agency as opposed to adopting a laissez-faire attitude and evading remedying the obstacles.

Beyond teachers’ agency, due to the mediating role of the contextual difficulties on student-teachers’ intention to perform genre instruction and their actual teaching practices, the concerted efforts from institutional administrators, curriculum and material developers as well as policymakers are needed to create a more supportive environment for teacher professional development regarding GBA. In providing experiential learning opportunities under the joint supervision of universities and field schools, teacher education programs should have more preparatory work to inform prospective teachers about the professional teaching methods, teaching settings, and student needs, helping them fit into the local ecology. Also, chances for professional development, like the tutorial, apprenticeship, and community-based learning should be taken into account, affording students to bring their teaching and learning enthusiasm into full play.

Understandably, this study is not free from limitations. Due to the practical constraints, the current study mainly depended on data from student-teachers’ semi-structured interviews and collected artifacts, without including other data sources such as classroom observation and stimulated recall, which may yield some firsthand information about the local teaching and learning contexts and the participants’ actual instructional practice. Accordingly, future studies can include data triangulation to provide a holistic understanding of student-teachers’ GBWI experience in the Chinese secondary EFL contexts. Another limitation is that we only investigated eight participants from normal universities, which consequently restricts the generalizability of our study. Further research can involve larger and more representative subjects (e.g., EFL student-teachers’ from different educational backgrounds in different areas) through large-scale surveys, so as to find the correlation of teacher cognition and practice with individual or contextual variables. Finally, this study investigated teachers’ knowledge and use of genre instruction within a short timeframe, failing to address the diachronic dynamics between the external teaching environment and internal teacher psychology. Therefore, based on the current finding of the interplay of the teacher knowledge and perceived difficulties in practicing genre pedagogies, researchers can also use longitudinal inquiry to trace how teacher cognition may develop through consistent reflection and whether the perceived external difficulties be solved during the instructional process.

## Data availability statement

The raw data supporting the conclusions of this article will be made available by the authors, without undue reservation.

## Ethics statement

The studies involving human participants were reviewed and approved by Southeast University. The patients/participants provided their written informed consent to participate in this study.

## Author contributions

CL contributed to develop the research ideas, collect and analyzed the data, and prepare for the manuscript. MC contributed to collect the data and revise the manuscript. Both authors contributed to the article and approved the submitted version.
