# Supplementary material for: A genre-based approach in the secondary school English writing class: Voices from student-teachers in the teaching practicum
Source: Front Psychol. 2022 Sep 6;13:992360. doi: 10.3389/fpsyg.2022.992360 (PMC9488108; doi:10.3389/fpsyg.2022.992360)
Supplement: Supplementary file 1 [file Table_1.DOCX]

Supplementary Material

# **Appendix A. Interview guide**

- 1. Basic information (e.g., teaching experience, education background, academic field and qualifications)

2. What is genre, in your view?

3. What do you think of drawing on the concept of genre to facilitate your teaching in the writing class?

4. What are the pedagogical focus of genre-based writing instruction (GBWI)?

5. Please describe a typical GBWI lesson that you have given.

6. What do you find most challenging about implementing GBWI in your classes? And why?
